# Records of *the Organon* and Materia Medica Club of the Bay Cities of California

**Published:** 1894-10

**Authors:** W. E. Ledyard

**Affiliations:** Secretary


W. E. Ledyard.

---

### RECORDS OF *THE ORGANON* AND MATERIA MEDICA CLUB OF THE BAY CITIES OF CALIFORNIA.

Members of Club.—Drs. J. M. Selfridge, Ormiston Swayze, A. McNeil, Wm. Bœricke, George J. Augur, C. M. Selfridge, M. T. Wilson, W. E. Ledyard.

### July 26th, 1894.

Dr. J. M. Selfridge reported a case of *neuralgia* of the *left* side of the head, in a woman aged forty-six, in whom the menses had been absent for several months, but with nose-bleed as vicarious menstruation. The patient at first described the pains as *coming on suddenly*, lasting for an indefinite time, and *leaving suddenly*. For this indication she received *Belladonna*, but without relief. In taking the case again, the patient insisted that, although the pains *came suddenly, they left gradually*. Now she received *Pulsatilla*, but again without relief. Finally she stated that the pains were *worse about* 1 A. M.; that they were like *hot* needles, and were relieved by heat. *Arsenicum*$^{100m}$, one dose dry, cured.

Dr. Swayze reported the following extremely interesting case:

Woman, aged forty-five; married; three children.

(History: mother had eczema, after the birth of the above.) Eczema after each labor. Ophthalmia neonatorum when eighteen; purulent, from inoculation by a strange child. Treated by *Arg-nit.* and *Bluestone.*

Disappeared; then amaurosis, iridectomy; sight fair.

Then had her first child, at twenty-four; eczema between fingers, following labor; eczema suppressed, followed by another attack of ophthalmia; ophthalmia treated locally; then uterine neuralgia and profuse menses, with relief to eye symptoms.

Second labor, three years later; similar symptoms.

Third labor, eight years later; similar symptoms.

Five years later; menopause; eye symptoms greatly aggravated; cornea opaque; a staphylomatous condition; menses absent eight months; menses reappear, with relief to all eye symptoms.

Present condition: Eyes dry (very); *stinging, burning, itching;* ameliorated by *cold* water and by application of *cold* milk; same sensation in eyes as in old eczema. On July 28th, after instilling *Atropine,* followed speedily by nausea, vomiting, pressure and throbbing in head, with delirious mutterings; answering when spoken to, but lapsing into delirium immediately, the doctor believes *Apis* to be the indicated remedy, but so far has had no opportunity to administer it.

AUGUST 3D, 1894.

Sections 1 to 12 of *The Organon* were read and discussed.

The following cases were reported: two cases treated by Dr. Chapman.

One began as a wen on the scalp; developed into an *epithelioma,* which went on to suppuration and ulceration, discharging pus, with *hot stabbing midnight pains;* great prostration, and other marked *Arsenicum* symptoms. *Arsenicum*$^{100m}$ gave speedy relief.

The other, *a scirrhus tumor* of left breast, with similar symptoms, ameliorated by same remedy, in same potency.

*August 6th,* Dr. Chapman writes: "The case of *epithelioma*

*of the scalp* has entirely recovered, and the *scirrhus of the breast* is progressing finely toward recovery."

Dr. McNeil—Case of a surgeon who contracted *diarrhœa* during the Mexican war, came for treatment, the diarrhœa being then of thirty-six years' standing. *Symptoms:* Stools involuntary—putrid—exhausting. Inability to take any food but corn bread, coffee, *fat meat*, and sweet potatoes. Indications were clear for *Arsenicum*, which he received in the 9th, increasing to the highest potency, as the action of the previous potency became exhausted. *Arsenicum* alone, cured in eighteen months, although there were, in the meantime, long intervals of freedom from diarrhœa.

Also case of a negro, aged fifty-eight. Diagnosed by Drs. Isham and Holland, of Louisville, Ky., as *enlarged prostate. Symptoms:* Dropsy—compelled to sleep in a chair, on account of dyspnœa; *had passed no urine*, except through the catheter, *for six months;* thirst for small quantities of water, and other symptoms indicating *Arsenicum*, which was given in single doses, commencing with the 200th potency, and gradually ascending the scale. *Began to pass urine naturally* in a few days. Dropsy soon disappeared, and in *seven months* he was ready to return to duty as a common laborer.

Dr. Augur—Girl who had a discharge of *mucus, excessively excoriating*, from the rectum, for *five years.* The discharge was attended with pain and *burning* in the back; menses: *scanty, short*, and *pale;* restless; puffy ankles. *Pulsatilla*$^{200}$ was given three times a day, for a week, and then stopped. In two weeks there was an improvement in every respect. Then a dose of *Puls.*$^{200}$ was given *once in ten days.*

Dr. Swayze—Case of *dental fistula*, which had been relegated to the knife. *Calc-fluor.*$^{30}$ caused a very profuse discharge from the fistula, and in six weeks cured it.

Also a case of *fistula in ano*, with oozing discharge, and suicidal tendency. The fistula had been cut open. *Rhus-tox.*$^{2}$ *cured.*

Dr. McNeil—While treating a case, cured a rupture without knowing it.

In discussing section 3 of *The Organon*, which relates to *Hygiene*, Dr. McNeil alluded to the *cravings of the sick*, and stated that he considered them in accordance with the above law, and allowed patients to have what they longed for in the line of food.

Dr. Wilson reported a case of diarrhœa in a child, with *craving for green corn*, which was allowed and the diarrhœa disappeared.

The Secretary reported a case where *raw peanuts* were permitted with relief.

Dr. McNeil—The *mental* symptoms are of very great importance. Hahnemann tells us in note to section 210 that "*Aconite* seldom or never effects a permanent cure, when the temper of the patient is quiet and even; or *Nux-vomica*, when the disposition is mild and phlegmatic; or *Pulsatilla*, when it is lively, serene, or obstinate; or *Ignatia*, when the mind is unchangeable, and little susceptible of either fear or grief."

### August 17th, 1894.

*The Organon* was read and discussed, from sections 13 to 20, inclusive.

Dr. McNeil, in discussing *section* 13, asked "what must be cut away?" He alluded to a case of *polypus*, removed by caustics and afterward by the wire ecraseur, by both allopaths and professed homœopaths. Operations were repeatedly performed. The child died after some months of such treatment. It is a very simple matter to remove a polypus, but *the taint to the system is what should be removed.* He compared the human system to three lines of works or fortifications, proceeding from within outwards. It would not be considered good generalship to drive the enemy from the outer to the inner fortification. Yet this is exactly what the old school does. Nature drives disease *from within out and from above downward.*

Dr. Swayze—What about "Orificial Surgery?" Let us settle that point before proceeding further. It is asserted by some that an eczematous eruption is actually removed from the system by cutting away the foreskin. Can enormously enlarged tonsils be cured by the indicated remedy? After removing the tonsils, a honey-comb condition remains.

Dr. McNeil—"When I was in Indiana, a girl of seventeen, who had a good training in vocal music, presented herself for treatment. She had had enlarged tonsils, which were removed by operation, on account of throat trouble, which continued, notwithstanding. She received *Hepar-sulph.* on the indication '*sensation of a splinter in the throat.*' This symptom is also found under *Alumina, Arg-nit., Dolichos., Nitr-ac., and Kali-carb.* The *Hepar* cured in about a year."

Dr. J. M. Selfridge: (who is a skillful surgeon) "I never removed a prepuce in my life." He reported the case of a little fellow; nervous, irritable, and fretful; frequently putting the hands to the prepuce. On examination, the prepuce, although not specially elongated, was not sufficiently dilated to expose the glans. On dilating the orifice of the prepuce, *hardened smegma* was found and removed; all further trouble disappearing.

Dr. C. M. Selfridge—Symptoms resembling *chorea* disappeared after removing the prepuce.

Dr. J. M. Selfridge reported the case of a young man *who had never exposed the glans, and yet had contracted gonorrhœa.* The *prepuce was dilated* in two months with the help of *Cocaine.*

He considers dilatation much better than amputation.

Dr. Augur—Elongated prepuce reacts on the system in a reflex way.

Dr. J. M. Selfridge—There is a possibility of the mucous membrane becoming very hard, but, even in that case, thinks dilatation will cure. As an example of reflex action, he referred to the *headaches from astigmatism* relieved by glasses. If a case of this kind (contraction of opening of prepuce) were taken in Hahnemann's way, he believes the orifice would dilate naturally.

Dr. McNeil—Neither headache nor kidney nor stomach trouble can be cured by taking into consideration only the part or organ complained of.

Dr. J. M. Selfridge referred to *several cases* of pneumonia, with *similar symptoms;* one had peculiarities calling for *Phosph.:* a second, with the *restlessness* characteristic of *Rhus,* was cured by that remedy. While the third, which had also *sticking* pains *from every movement,* with *great thirst, parched lips,* etc., found its curative in *Bryonia.*

Dr. McNeil reported a case to show that it is a mistake to base one's prescription on *one* symptom. The case was *puerperal metritis,* with *aggravation from the least movement. Bryonia* was given and failed. On examining the case more closely, the patient was found to be in a *profuse sweat without relief. Mercurius* relieved in six hours.

The leading characteristic of the nineteenth century is belief in the reign of law. In disease alone there is no belief in law, from an allopathic standpoint. The treatment of *cholera* illustrates this. The allopathic treatment of this disease is empirical. Concerning its homœopathic treatment, *Hahnemann* stated that *Camphor, Cuprum,* and *Veratrum-album,* according to indications, would cure. *Astronomy,* as an exact science, is on the same line with *Homœopathy. Ignatia* and *Nux-vomica* both contain *Strychnine.* Hahnemann has pointed out the difference between these two, in the spirit force. Chemical properties are not sufficient to show curative powers.

Dr. Swayze—Although the *Strychnine* is the same, the total is different.

Dr. J. M. Selfridge—The pharmacist can separate, but cannot re-unite. The vital power is gone.

Mr. Underwood—Why cannot the system absorb the spirit-like force from the tincture?

Dr. McNeil—The spirit-like vital force is sick; it *is* spirit-like, and the remedy for its removal must, in like manner, be immaterial.

Dr. J. M. Selfridge—The spirit-force has a greater analogy for the spiritual. *Calcarea-carb., Mercurius, Lycopodium, Silicea,* and *Carbo-vegetabilis, in crude form, give no symptoms.* The crude drug is not taken into the system as readily as the dynamized drug.

Dr. McNeil—The proving of *Natrum-muriaticum* is a good illustration. We all take *salt* in health, but, when sick, the *dynamized Natrum-mur*. will cure.

He referred to the case of a soldier, with a *craving* for *salt;* cured by the *dynamized Natrum-mur*. "The Vienna Provers' Union" found no effects from taking the crude Natrum-mur., and not until they took it in the highly potentized form were symptoms of the drug developed.

(Signed) W. E. LEDYARD,
*Secretary.*

